# Assessing the epithelial-to-mesenchymal plasticity in a small cell lung carcinoma (SCLC) and lung fibroblasts co-culture model

**DOI:** 10.3389/fmolb.2023.1096326

**Published:** 2023-03-03

**Authors:** Nilu Dhungel, Reneau Youngblood, Min Chu, Jennifer Carroll, Ana-Maria Dragoi

**Affiliations:** ^1^ Department of Molecular and Cellular Physiology, LSUHSC-Shreveport, Shreveport, LA, United States; ^2^ Feist-Weiller Cancer Center, INLET Core, LSUHSC-Shreveport, Shreveport, LA, United States; ^3^ Center for Emerging Viral Threats (CEVT), LSUHSC-Shreveport, Shreveport, LA, United States

**Keywords:** SCLC-small cell lung cancer, EMT-epithelial to mesenchymal transition, YAP1 (yes-associated) protein, EMT-TFs, EMT plasticity

## Abstract

The tumor microenvironment (TME) is the source of important cues that govern epithelial-to-mesenchymal transition (EMT) and facilitate the acquisition of aggressive traits by cancer cells. It is now recognized that EMT is not a binary program, and cancer cells rarely switch to a fully mesenchymal phenotype. Rather, cancer cells exist in multiple hybrid epithelial/mesenchymal (E/M) states responsible for cell population heterogeneity, which is advantageous for the ever-changing environment during tumor development and metastasis. How are these intermediate states generated and maintained is not fully understood. Here, we show that direct interaction between small cell lung carcinoma cells and lung fibroblasts induces a hybrid EMT phenotype in cancer cells in which several mesenchymal genes involved in receptor interaction with the extracellular matrix (ECM) and ECM remodeling are upregulated while epithelial genes such as E-cadherin remain unchanged or slightly increase. We also demonstrate that several core EMT-regulating transcription factors (EMT-TFs) are upregulated in cancer cells during direct contact with fibroblasts, as is Yes-associated protein (YAP1), a major regulator of the Hippo pathway. Further, we show that these changes are transient and reverse to the initial state once the interaction is disrupted. Altogether, our results provide evidence that tumor cells’ direct contact with the fibroblasts in the TME initiates a signaling cascade responsible for hybrid E/M states of cancer cells. These hybrid states are maintained during the interaction and possibly contribute to therapy resistance and immune evasion, while interference with direct contact will result in slow recovery and switch to the initial states.

## Introduction

The surrounding tumor stroma represents a critical microenvironment for cancer cells to grow and initiate metastasis. Tumor microenvironment (TME) consists of different cell types (including fibroblasts, immune cells, and epithelial cells), vasculature, and extracellular matrix (ECM) including collagens, fibronectin, elastin, and proteoglycans (Anderson and Simon, 2020). There is a constant crosstalk between the tumor cells and TME, which provides mechanisms and signaling for tumors to survive and progress (Pickup et al., 2014; Valenti et al., 2017; Henke et al., 2019; Hinshaw and Shevde, 2019). Cancer-associated fibroblasts (CAFs) are one of the major components of TME. Numerous studies have shown that CAFs secrete various factors (including growth factors, cytokines, and enzymes) that promote stemness, angiogenesis, ECM remodeling, immune evasion, and therapeutic resistance in cancer cells (LeBleu and Kalluri, 2018; Liu et al., 2019; Sahai et al., 2020). Equally, tumor cells can induce and maintain CAFs in their activated state creating a feed-forward loop (Nair et al., 2017; Valenti et al., 2017; Fiori et al., 2019; Wang et al., 2022). Thus, CAFs and cancer cells have a mutually beneficial and reciprocal relationship that abets cancer progression.

Acquisition of aggressive traits by cancer cells such as an invasive phenotype and chemoresistance represent hallmark features of metastatic cancer (Hanahan and Weinberg, 2011; Valastyan and Weinberg, 2011; Vanharanta and Massague, 2013; Welch and Hurst, 2019). The metastatic process is initiated by the epithelial-to-mesenchymal transition (EMT) in cancer cells. The fundamental principle of EMT is the activation of a molecular program that drives the acquisition of a mesenchymal phenotype by the epithelial cells (Zhang and Weinberg, 2018; Dongre and Weinberg, 2019). During EMT, cells start to lose their epithelial markers and gain mesenchymal markers, a process driven by the high expression of a core set of EMT-activating transcription factors (EMT-TFs), the ZEB, SNAI, and TWIST families (Peinado et al., 2007; Zhang et al., 2015; Simeone et al., 2019; Stemmler et al., 2019). Cells displaying a mesenchymal phenotype lose cell-cell junctions, become invasive, and migrate from the original epithelium to disseminate to secondary sites. Because EMT-TFs have pleiotropic functions, EMT also is associated with escape from apoptosis, acquisition of stemness properties, therapy resistance, and immune evasion (Caramel et al., 2018; Brabletz et al., 2021). A dynamic transition between epithelial and mesenchymal phenotypes is responsible for EMT plasticity and favorably contributes to cancer progression. Thus, during metastasis cancer cells exist in a multitude of intermediate hybrid phenotypes that retain both epithelial and mesenchymal markers (Jolly et al., 2015; Nieto et al., 2016; Brabletz et al., 2018; Jolly et al., 2019; Deshmukh et al., 2021). Single cell RNAseq analysis highlighted that, for example, in response to prolonged TGFβ1 treatment cells undergo multiple intermediate EMT states, culminating with cadherin switch at day 8 post-treatment (Deshmukh et al., 2021). How this spatial and temporal plasticity is achieved during tumor progression remains a key question of EMT and cancer metastasis.

For several cancer types including hepatocellular carcinoma, pancreatic cancer, breast cancer, lung cancer, melanoma, and colon cancer it has been demonstrated that tumor cells interaction with CAFs promotes EMT and the metastatic phenotype (Kubo et al., 2016; Domen et al., 2021; Shelton et al., 2021; Ahmad Zawawi and Musa, 2022; Ando et al., 2022; Hu et al., 2022). Direct and indirect mechanisms have been shown to influence tumor metastasis by CAFs: i) ECM remodeling by secretion of collagens, fibronectin, and proteoglycans (Scott et al., 2019); ii) paracrine communication through exosomes, growth factors and cytokines that promote stemness and metastasis, such as: transforming growth factor β - TGFβ, Chemokine (CC motif) ligand 2 (CCL2), Interleukin-6 (IL-6), Hepatocyte growth factor (HGF), Osteopontin (OPN) and Stromal cell-derived factor 1 (SDF-1) (Linares et al., 2020); iii) direct contact with cancer cells and facilitated invasion and select gene transcription (Yamaguchi and Sakai, 2015; Liu et al., 2019).

Previous studies revealed that squamous carcinoma cells require direct contact with CAFs to invade the ECM in a 3D co-culture system (Gaggioli et al., 2007). In addition, in a prostate cancer co-culture model, fibroblasts promote directional cancer cells migration by organizing the fibronectin matrix (Erdogan et al., 2017). Direct cancer cells-CAFs interaction also enhances the invasion of lung carcinoma cells in a 3D co-culture system (Otomo et al., 2014) and *in vivo* (Duda et al., 2010). Furthermore, TGFβ1 enhances the dissemination of colon carcinoma cells to the liver through attachment to CAFs (Gonzalez-Zubeldia et al., 2015). Gastric carcinoma studies showed that CAFs promote a strong invasive phenotype when in direct contact with scirrhous gastric carcinoma cells (Yamaguchi et al., 2014; Satoyoshi et al., 2015), whereas CAFs conditioned media did not, emphasizing the importance of direct contact between cancer cells and fibroblasts in invasion (Satoyoshi et al., 2015). Conditioned media from breast cancer cells and fibroblasts direct co-cultures increased cancer cells’ metastatic potential while conditioned media from homotypic cultures had little effect, indicating the role of direct contact between cancer cells and fibroblasts in the release of soluble factors and paracrine signaling as well (Stuelten et al., 2010). The molecular mechanisms by which CAFs directly stimulate cancer cells migration are still poorly understood. A heterotypic and mechanically active E-cadherin/N-cadherin interaction has been previously described that enables cancer cells invasion (Labernadie et al., 2017). Recently, the direct interaction was shown to involve integrin-α5β1 (ITGA5/ITGB1) on cancer cells and fibronectin assembled on the surface of CAFs (Miyazaki et al., 2019; Miyazaki et al., 2020). Importantly, distinct transcriptome profiles have been detected in cancer cells depending on whether they can directly contact CAFs or not in co-culture models (Camp et al., 2011). Here, we use a small cell lung carcinoma (SCLC) and lung fibroblast *in vitro* model to investigate the effects of direct vs. indirect interaction on gene reprogramming in cancer cells.

We demonstrate that specifically upon direct contact with fibroblasts, cancer cells undergo profound reprogramming and develop a partial EMT phenotype in which EMT-inducing growth factors, as well as ECM remodeling proteins, are highly upregulated. Moreover, this transcriptome reprogramming was accompanied by significant increase in ZEB1, ZEB2 and TWIST2 EMT-TFs in cancer cells. Importantly, Yes-associated protein (YAP1), a critical component of the Hippo pathway and co-activator of genes related to EMT and cell growth (Akrida et al., 2022), was also highly upregulated in cancer cells upon direct contact with fibroblasts. The cancer cells reprogramming by fibroblasts was transient and upon separation from fibroblasts cancer cells slowly reverted to their original more epithelial phenotype. The results reveal the importance of direct contact between cancer cells and fibroblasts in inducing a hybrid and transient EMT phenotype in cancer cells.

## Results

### Cancer cells attach to and migrate with fibroblasts in co-culture

**FIGURE 1:**
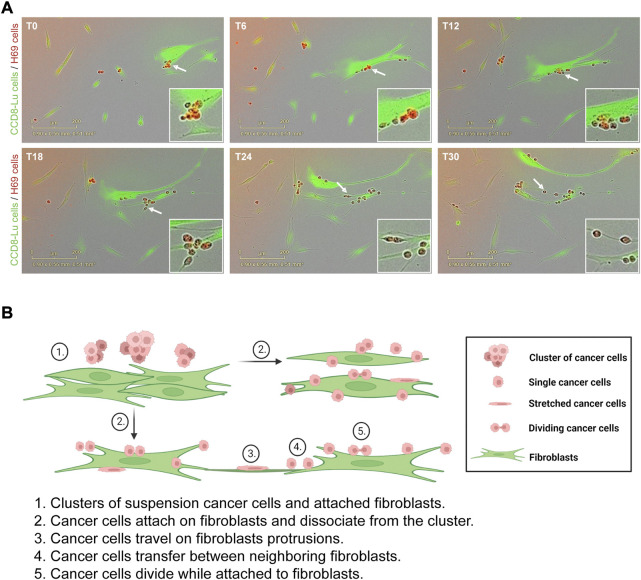
H69 cancer cells attach and travel on fibroblasts. **(A)** Representative micrographs of H69 cancer cells (red) cells interacting with CCD8 fibroblasts (green). Six time points (T0, T6, T12, T18, T24 and T30) are presented from a 72 h acquisition. Insets show an area of interaction at different time points (white arrow). **(B)** Model of cancer cells interaction with fibroblasts. The model depicts different states of interaction (1–5).

To evaluate the effects of direct contact between cancer cells and fibroblasts we used a model of direct and indirect co-culture of H69 small-cell lung carcinoma cells with CCD8 normal lung fibroblasts. In direct co-culture, both cell lines are grown in physical contact for 72 h. In the indirect co-culture, cancer cells and fibroblasts are grown separated by a permeable insert thereby they only communicate *via* soluble factors. H69 are suspension cells and grow in floating cell clusters while CCD8 are adherent cells. To observe how the cancer cells and fibroblasts interact with each other, we co-cultured green fluorescent-labelled CCD8 cells with red fluorescent-labelled H69 cells for 72 h. Live-cell imaging was used to monitor the interaction hourly for 3 days (Figure 1A, Supplementary Movies S1, S2). Continuous live imaging showed that H69 cells adhere to CCD8 fibroblasts within a few hours and remained attached for long periods of time. Several scenarios were observed: i) cancer cells attach and migrate together with fibroblasts on the fibroblasts’ cell surface or fibroblasts’ protrusions; ii) cancer cells transfer from one fibroblast to another and between dividing fibroblasts; iii) cancer cells divide and proliferate while they are still attached to the fibroblasts; iv) contact between cancer cells clusters and fibroblasts induce separation of cancer cells in the cluster. Importantly, we also noticed that some of the cancer cells are temporarily stretched as the fibroblasts migrate, indicating that cancer cells experience mechanical tension as they migrate with the fibroblasts. These scenarios (Figure 1B) suggest that the direct interaction between cancer cells and fibroblasts is complex and allows cancer cells to seize fibroblasts for invasion while possibly going through rounds of mechanical stretching.

### Direct interaction with fibroblasts triggers transcriptional reprogramming in cancer cells

**FIGURE 2:**
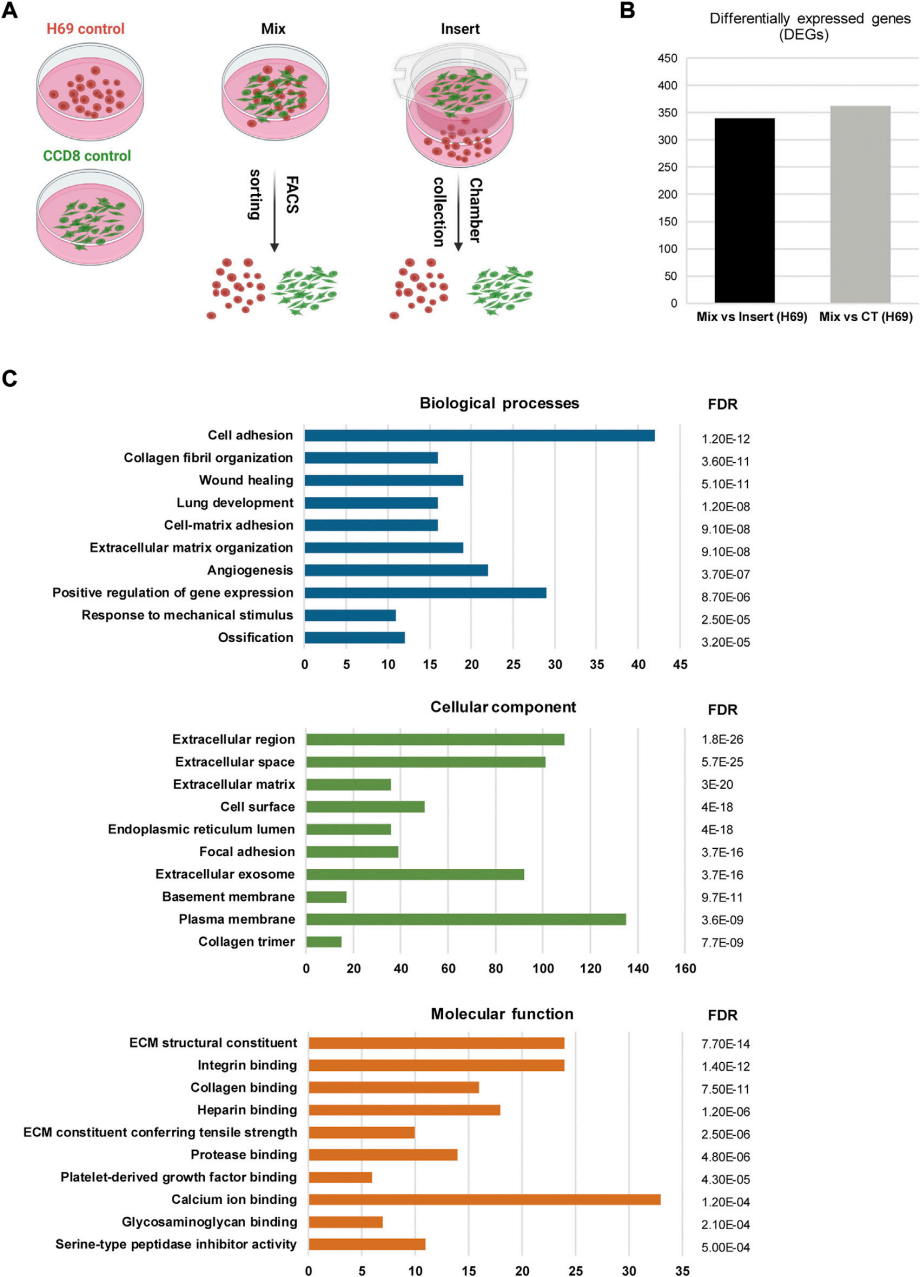
Gene analysis in H69 cells during direct interaction. **(A)** Experimental set-up for direct and indirect interaction between fluorescently labeled H69 cancer cells and CCD8 fibroblasts. **Control**, cells were grown in separate dishes. **Mix**, cells were co-cultured in direct contact before FACS sorting. **Insert**, cancer cells and fibroblasts were grown in close proximity and separated by an insert. Cells were cultured in their respective conditions for 72 h before RNA extraction. **(B)** Differentially expressed genes (DEGs) in H69 cells with 2-fold increase (log_2_FC > 1) or decrease (log_2_FC < −1) in gene expression. **Mix *vs*. Insert** shows the number of genes regulated during direct contact compared to indirect contact. **Mix *vs*. CT** shows the number of genes regulated during direct contact compared to control conditions. **(C)** Gene ontology analysis of DEGs specifically regulated in direct contact (Mix) compared to indirect contact (Insert). The top ten enriched GO terms are presented for Biological processes, Cellular component, and Molecular function. The number of genes enriched in each term is shown on the *x*-axis. FDR value is shown for each enriched term.

The induction of EMT signatures in cancer cells following interaction with fibroblasts has been reported previously in other cancers (Giannoni et al., 2010; Camp et al., 2011). Thus, we sought to investigate gene expression in cancer cells and fibroblasts in the context of direct and indirect contact. For this, we cultured H69 cancer cells under three different conditions for 72 h (Figure 2A): i) monoculture, in which both cells lines are cultured alone (Figure 2A, Control); ii) indirect co-culture conditions, in which CCD8 cells are cultured in an insert separated from the H69 cells by a 1.0 µm pore membrane (Figure 2A, Insert); iii) direct co-culture conditions (Figure 2A, Mix), in which H69 and CCD8 can contact each other. Both cell lines were fluorescently labeled prior to co-culture to allow for subsequent segregation of the two populations. Following 72 h incubation, CCD8 fibroblasts and H69 cancer cells were collected from Control (CT), Insert and Mix conditions after FACS sorting. Microarray analysis was performed to identify differentially expressed genes (DEG) in both cell lines. Based on the analysis setting (FDR <0.1, fold change < −2 or >2) a total of 362 genes were significantly changed under direct co-culture conditions in H69 cancer cells as compared to control conditions (Figure 2B, Mix vs. CT, Supplementary Table S1). Remarkably, the majority of these genes (339 of 362 genes) were differentially regulated in direct vs. indirect interaction (Figure 2B, Mix vs. Insert, Supplementary Tables S2, S3), demonstrating that the direct contact between cancer cells and fibroblasts is likely the main driver of transcriptional reprogramming in cancer cells. Notably, under these analysis settings, only 19 genes were differentially regulated in CCD8 cells in direct contact with cancer cells and only one gene (IL32) was differentially regulated in CCD8 in indirect contact with cancer cells (Supplementary Tables S3–S5). This difference in gene transcription regulation suggests that either fibroblasts’ reprogramming is more subtle, thus below the limit of detection under these settings or, the changes are not detected in the same time frame (72 h). Importantly, the vast majority of DEG in H69 cells were upregulated after direct contact (Figure 2B, Mix vs. Insert). These results highlight the major role direct interaction with fibroblasts plays in gene reprogramming in cancer cells.

To obtain further insight into the function of the 339 identified DEGs in H69 cells (Mix vs. Insert), the Database for Annotation, Visualization and Integrated Discovery (DAVID) (Jiao et al., 2012; Sherman et al., 2022) and Kyoto Encyclopedia of Genes and Genomes (KEGG) were used to perform Gene Ontology (GO) and pathway enrichment analyses, respectively (Supplementary Tables S7, S8). The GO analysis indicated that 1) for biological processes (BP), DEGs were significantly enriched for cell adhesion, collagen fibril organization, cell-matrix organization, wound healing, and angiogenesis (Figure 2C, Biological processes); 2) for cellular component (CC), DEGs were significantly enriched for extracellular region, cell surface, focal adhesion and extracellular exosomes (Figure 2C, Cellular component); 3) for molecular function (MF), DEGs were enriched in ECM structural constituent, integrin binding, collagen binding, and ECM constituent conferring tensile strength (Figure 2C, Molecular function).

The GO analysis conclusively shows that following direct interaction with the fibroblasts H69 cells undergo phenotypic changes that favor interaction with the ECM and cellular migration. Among the highest upregulated proteins in H69 cells were transgene (TAGLN), fibronectin (FN1), transforming growth factor, beta-induced (TGFBI), and vimentin (VIM), all markers of EMT (Liberzon et al., 2015) (Table 1). This is suggestive of EMT reprogramming towards a more aggressive and invasive phenotype.

**TABLE 1:** Differentially expressed genes in H69 cells.

Gene Symbol	Fold Change	H69_MIX Avg (log2)	H69_1NS Avg (log2)	p-val	FDR p-val	Public Gene IDs	Description
FNl	1201.53	14.61	4.38	2.32E-18	2.79E-13	NM_001306129	fibronectin 1
SPARC	346.1	13.71	5.27	6.08E-18	2.79E-13	NM_001309443	secreted protein, acidic,cysteine-rich (osteonectin)
TAGLN	180.17	13.67	6.17	6.17E-18	2.79E-13	NM_001001522	transgelin
VIM	537.77	15.22	6.15	1.29E-17	4.07E-13	NM_003380	vimentin
DCN	418.66	14.81	6.1	1.50E-17	4.07E-13	NM_001920	decorin
TGFBI	884.64	14.69	4.91	2.31E-17	5.22E-13	NM_000358	transforming growth factor, beta-induced, 68kDa
SERPINEl	221.07	13.41	5.62	1.07E-16	2.07E-12	NM_000602	serpin peptidase inhibitor member 1
MFGE8	199.45	13.61	5.97	1.35E-16	2.29E-12	NM_001114614	milk fat globule-EGF factor 8 protein
IGFBP3	836.21	13.86	4.15	1.05E-15	1.58E-11	NM_000598	insulin like growth factor binding protein 3
COL6A3	119.51	11.53	4.63	1.23E-15	1.67E-11	NM_004369	collagen, type VI, alpha 3

Top 10 DEGs, in H69 cells after interaction with fibroblasts are shown.

### Critical EMT regulators are enriched in cancer cells during direct contact with fibroblasts

Interestingly, H69 are upregulating genes involved in ECM deposition and organization during direct contact with fibroblasts (Table 2), suggesting that cancer cells themselves can be an active and important component of ECM remodeling. Indeed, cancer cells that acquired the ability to synthesize their own ECM proteins and ligands are able to escape proliferative suppression to grow and survive in hostile environments and become highly metastatic (Zahir and Weaver, 2004; Naba et al., 2012; Naba et al., 2014). The KEGG pathways analysis showed that DEGs involved in focal adhesion, proteoglycans in cancer, ECM-receptor interaction, and PI3K-Akt signaling are significantly enriched (Table 2). This suggests that following direct contact with fibroblasts, the production of tumor cell-derived ECM increases and might dictate the metastatic potential of the cancer cells.

**TABLE 2:** KEGG pathways enrichment in H69 cancer cells.

Pathway Name	Count	%	FDR	Genes
Focal adhesion	25	7.4	2.40E-09	ITGB1, LAMA2, ITGB5, SHC1, THBS2 , THBS1 , EGFR, MYLK, CCND1, CAPN2 , PDGFRA , CAV2 , ITGA3, ITGA2, CAV1 , FN1, VEGFC , COL 1A1, COL 1A2, COL6A2 , COL6A 1, COL6A3 , ITGA5, MYL9, VCL
Proteoglycans in cancer	23	6.8	7.60E-08	ITGB1, TGFB1 , ITGB5, CAV2 , LUM, ITGA2, MMP2 , CAV1 , WNT5A , FN1, MSN, FGF2, THBS1 , DCN, EGFR, COL 1A1, COL 1A2, CCND1, FAS, TIMP3 , ITGA5, TLR4 , CD44
ECM-receptor interaction	15	4.4	4.80E-07	ITGB1, LAMA2, ITGB5, ITGA3, ITGA2, FN1, THBS2 , THBS1 , COL 1A1, COL 1A2, COL6A2 , COL6A 1, COL6A3 , ITGA5, CD44
AGE-RAGE signaling pathway in diabetic complications	15	4.4	2.00E-06	EGR1, TGFB1 , STAT1 , MMP2, SERPINE1 , FN1, VEGFC , F3, TGFBR2 , COL 1A1, COL3A1 , IL6, COL 1A2, CCND1, CCL2
Pl3K-Akt signaling pathway	27	8	2.70E-06	ITGB1, LAMA2, ITGB5, LPAR1, THBS2 , FGF2, THBS1 , EGFR, FGF7 , CCND1, PDGFRA , ANGPT1 , ITGA3, ITGA2, FN1, VEGFC, OSMR, GNG12 , COL 1A1, IL6, COL 1A2, COL6A2 , COL6A1 , COL6A 3, ITGA5 , IL7R, TLR4
Hypertrophic cardiomyopathy	12	3.5	1.60E-04	ITGB1, IL6, TGFB1 , TPM4 , LAMA2 , ITGB5 , ITGA3, TPM2 , ITGA2, TPM1 , LMNA , ITGA5
Regulation of actin cytoskeleton	18	5.3	2.00E-04	ITGB1, PDGFRA , ITGB5, ITGA3, ITGA2, LPAR1, FN1, MSN, GNG12, FGF2, EGFR, MYLK, DIAPH2 , FGF7, BDKRB1 , ITGA5, MYL9, VCL
Complement and coagulation cascades	11	3.2	4.60E-04	SERPINB2 , CFH, C1S, SERPINE1 , SERPING1 , BDKRB1 , PLAT, TFPI, CLU, F3, F2RL2
Pathways in cancer	28	8.3	9.20E-04	ITGB1, LAMA2 , EPAS1, LPAR1 , FGF2, EGFR, FGF7, EDNRA, CCND1, BDKRB1 , HES1 , PDGFRA, TGFB1, MMP1, ITGA3, STAT1 , ITGA2, MMP2, WNT5A , FN1, V EGFC, GNG12 , TGFBR2 , RUNX 1, IL6, FAS, IL6ST, IL7R
Human papillomavirus infection	21	6.2	9.20E-04	ITGB1, LAMA2 , ITGB5 , STAT1 , ITGA3, ITGA2, WNT5A , FN1, THBS2 , THBS1 , EGFR, HLA-E, COL 1A1, COL 1A2, CCND1, COL6A2 , COL6A1 ,FAS, COL6A3 , HES1, ITGA5

Top 10 most enriched KEGG, pathways in H69 cells are shown.

**FIGURE 3:**
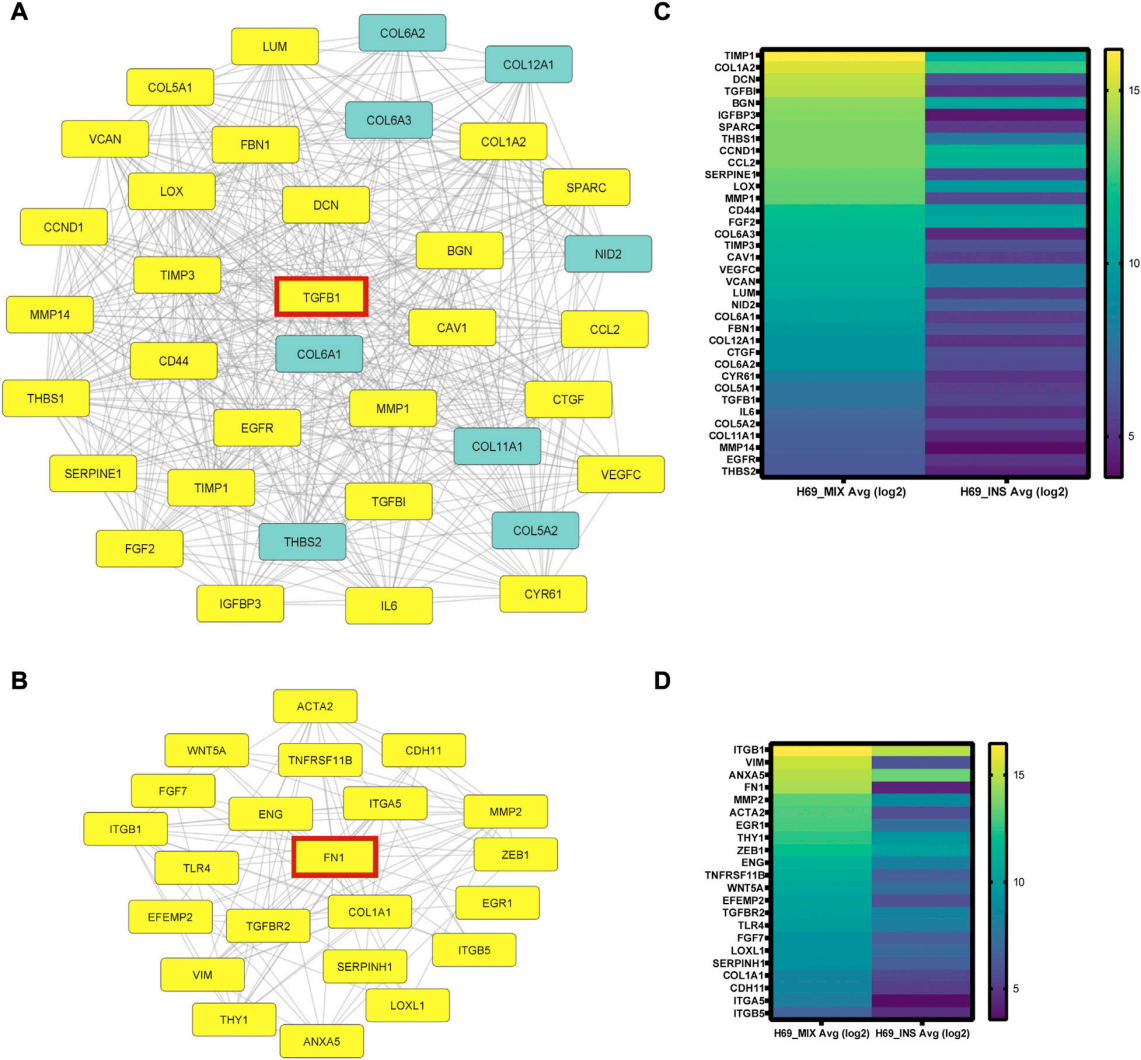
PPI network analysis using STRING online database and Cytoscape software. **(A-B)** Cytoscape MCODE was used to identify central nodes within the DEGs. In total 7 central nodes were identified. Cluster 1 **(A)** PPI network has 36 nodes and 436 edges. In yellow are the direct interactors of TGFB1, one of the DEGs in the cluster. Cluster 2 **(B)** PPI network has 22 nodes and 102 edges. In yellow are the direct interactors of FN1, a central DEG of the cluster. **(C-D)** Heat-map analysis of genes in cluster 1 **(C)** and cluster 2 **(D)** was completed using Prizm software and gene expression analysis provided by Transcriptome Analysis Console (TAC) from three independent experiments.

To analyze the protein-protein interaction (PPI) of the 339 genes differentially regulated in H69 during direct contact with the CCD8 cells, STRING online database and Cytoscape software were used to construct the PPI network. In total 339 genes were enrolled in the PPI network, and a high confidence score (>0.700) was applied. The PPI network shows a high degree of interconnectivity and includes 682 edges (PPI enrichment *p*-value <1.0e-16) (Supplementary Figures S1, S2). We used Cytoscape MCODE to cluster core genes which revealed 7 central nodes (Supplementary Table S9). Within the 7 central nodes, the two highest-scored clusters contained 36 nodes with 436 edges (score = 24.914) and 22 nodes with 102 edges (score = 9.714) respectively (Figures 3A, B). Interestingly, both clusters centered on key EMT inducers, TGFB1 in cluster 1 (Figure 3A) and FN1 in cluster 2 (Figure 3B) and their direct interactors (Figures 3A, B, highlighted in yellow). Heat-map analysis of the genes in clusters 1 and 2 revealed upregulation of EMT inducers such as TIMP-1, LOX, CTGF, CYR61, THBS1, and THBS2 in cluster 1 (Figure 3C) and EMT markers such as ZEB1, VIM, and ACTA2 in cluster 2 (Figure 3D). Taken together, our results show remarkable reprogramming in cancer cells during direct contact with the fibroblasts, suggesting that these cells undergo major transcriptional changes that sustain EMT and cancer progression.

### A hybrid EMT phenotype is induced in cancer cells during interaction with fibroblasts

**FIGURE 4:**
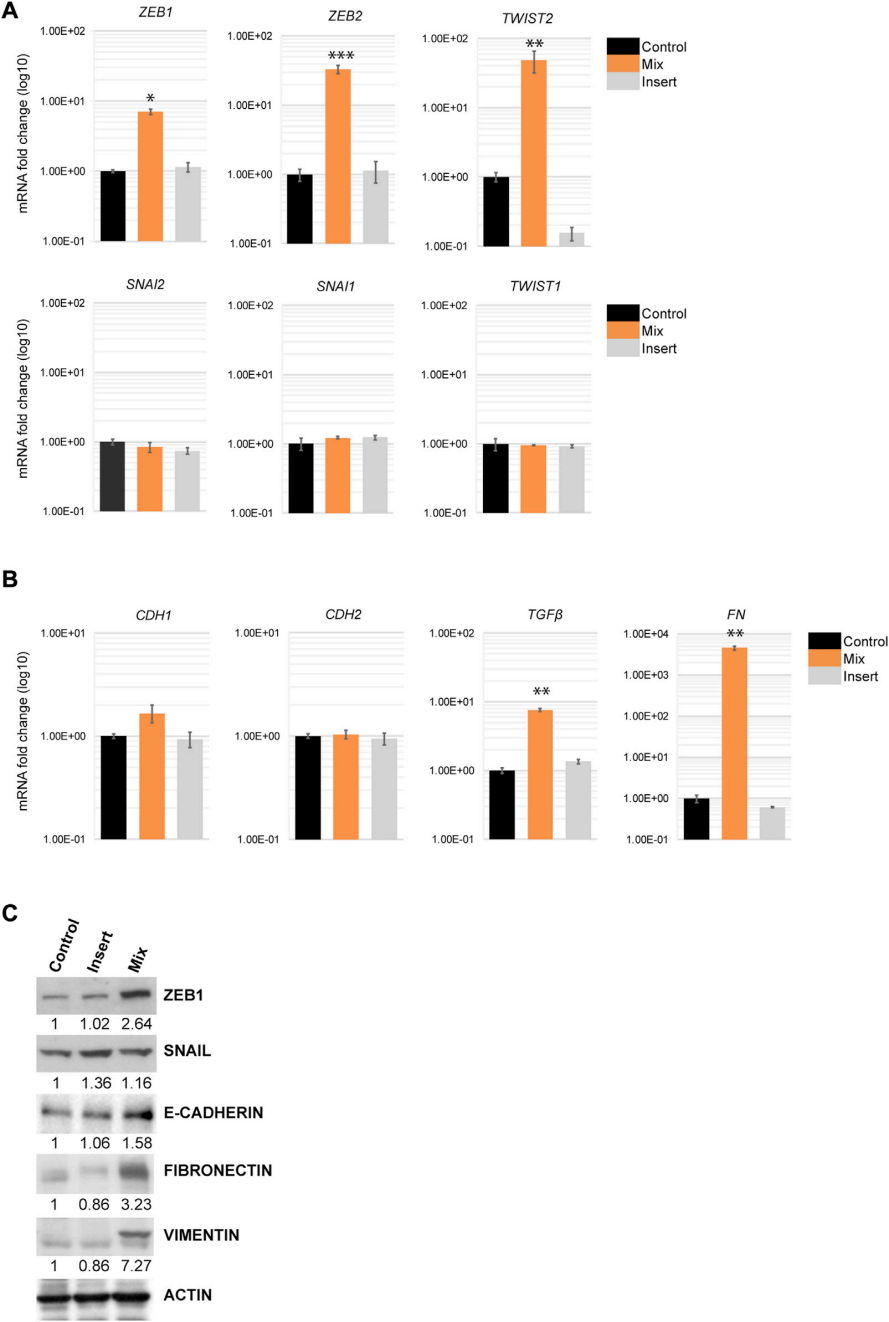
EMT markers regulation in H69 cancer cells during direct contact with CCD8 cells. **(A)** Gene expression for members of three EMT-TFs families was evaluated by qPCR in **Control**, **Mix** and **Insert** conditions in H69 cancer cells. mRNA levels are presented as fold increase (log10). Graphs are representative experiments from 4 to 6 independent repeats. **(B)** Epithelial and mesenchymal markers gene expression was examined by qPCR in H69 cells under the same conditions as in **(A)**. Graphs are representative experiments from 4 to 6 independent repeats. The significance of differences between **Control** and **Mix** conditions was determined by Student’s *t*-test (*, *p* < 0.05, **, *p* < 0.01, ***, *p* < 0.001). **(C)** Protein expression of ZEB1, SNAIL, E-cadherin, Fibronectin, and Vimentin was determined by Western blot. Actin was used as the loading control. Densitometry analysis was performed using ImageJ software.

Because EMT-TFs control multiple markers involved in EMT progression by repressing epithelial markers and activating mesenchymal markers, we next examined the transcriptional levels of the EMT-TFs in H69 cells that were in direct and indirect contact with fibroblasts. Quantitative PCR (qPCR) analysis and Western blot analysis of H69 cells cultured under different conditions confirmed that the level of EMT-TFs ZEB1, ZEB2, and TWIST2 were specifically upregulated in H69 cells in direct contact with CCD8 (Figures 4A, C, Mix) but not in cells in indirect contact (Figures 4A, C, Insert). Conversely, SNAI family and TWIST1 levels were not affected by the interaction. These results show a certain degree of specificity in EMT-TFs regulation during direct interaction. Despite the upregulation of specific EMT-TFs, E-cadherin, a marker of the epithelial phenotype in cancer cells was not downregulated (Figures 4B, C). Similarly, N-cadherin expression, a mesenchymal marker, did not change, suggesting that the E-cadherin/N-cadherin switch did not occur in H69 cells. Still, EMT inducers and mesenchymal markers such as TGFβ1, fibronectin, and vimentin are upregulated in H69 cells during direct contact (Figures 4B, C, Mix) indicating a hybrid E/M phenotype. Interestingly, OVOL1, a transcription factor of epithelial lineage (Saxena et al., 2022) was also upregulated in H69 cells (Supplementary Figure S3). This suggests that the ZEB1/OVOL1 axis, controlled by the Zeb1:OVOL1 ratio and their reciprocal inhibition (Roca et al., 2013; Jia et al., 2015), might be responsible for the partial EMT we observe in H69 cells during interaction with fibroblasts. Altogether, these results point to cancer cells undergoing a partial EMT in which cells maintain epithelial markers while upregulating expression of a subset of EMT-TFs and genes involved in the ECM remodeling and focal adhesion.

### Cancer cells upregulate YAP1 pathway during direct contact with fibroblasts

**FIGURE 5:**
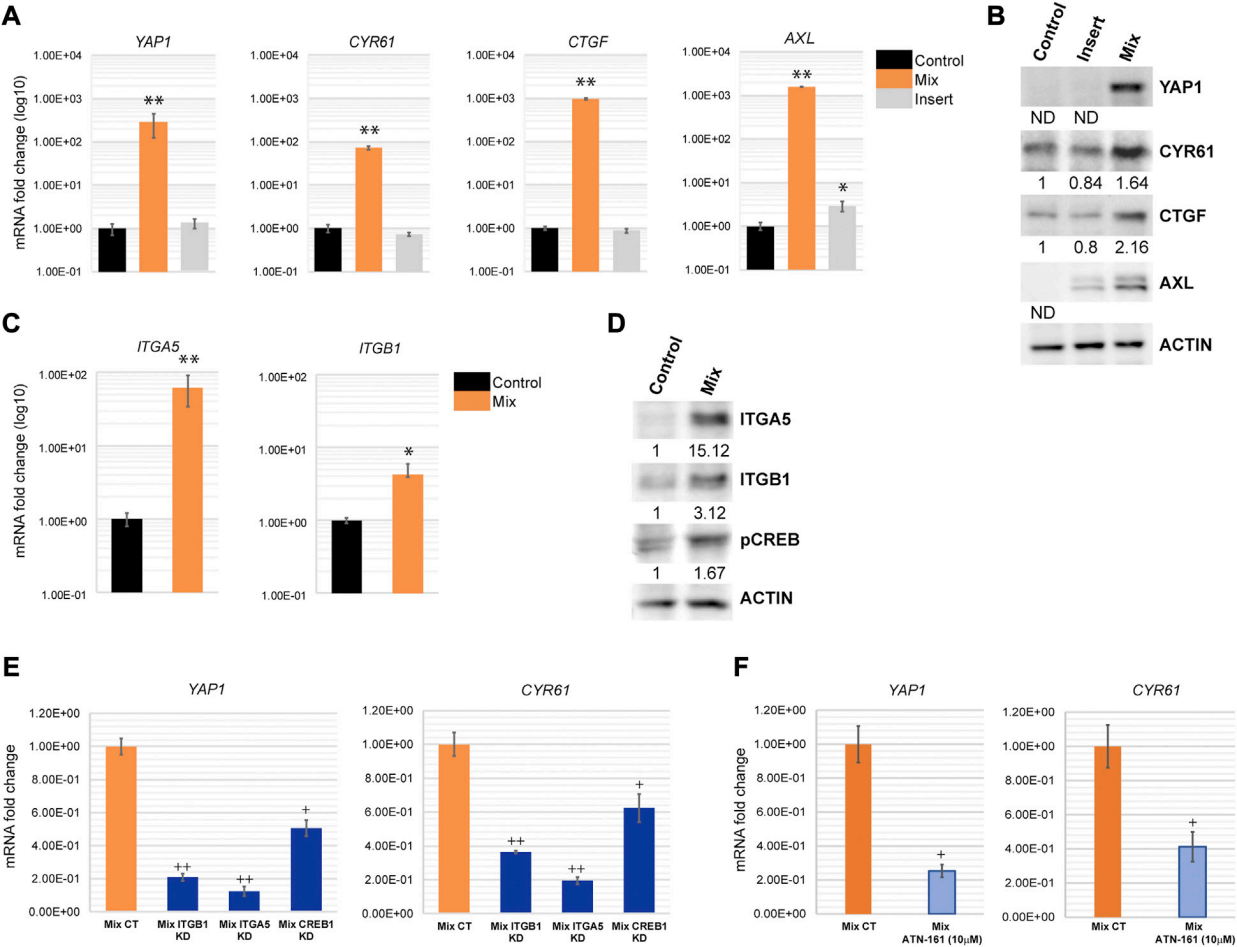
YAP1 is transcriptionally activated in H69 cells during direct contact with fibroblasts. **(A)** Relative mRNA levels of YAP1 and its targets *CYR61, CTGF* and *AXL* were determined by qPCR. mRNA levels are presented as fold increase (log10). Graphs are representative experiments from 4-6 independent repeats. **(C)** mRNA levels of *ITGA5* and *ITGB1* were determined by qPCR. mRNA levels are presented as fold increase (log10). Graphs are representative experiments from three independent repeats. **(A, C)** The significance of differences between **Control** and **Mix** conditions was determined by Student’s *t*-test (*, *p* < 0.05, **, *p* < 0.01, ***, *p* < 0.001). **(B, D)** Protein expression of YAP1, CYR61, CTGF, ITGA5, ITGB1, and pCREB was determined by Western blot. Actin was used as the loading control. Densitometry analysis was performed using ImageJ software. **(E)** Relative mRNA levels of *YAP1* and *CYR61* were determined by qPCR in H69 cells mock-transfected (**Mix CT**) or siRNA transfected with duplexes targeting ITGB1 (**Mix ITGB1 KD**), ITGA5 (**Mix ITGA5 KD**), and CREB (**Mix CREB KD**) and co-cultured with fibroblasts for 48 h. **(F)** Relative mRNA levels of *YAP1* and *CYR61* were determined by qPCR in H69 cells in DMSO-treated (**Mix CT**) or ATN-161-treated (10 μM) (**Mix ATN-161**) co-culture conditions. H69 and CCD8 were co-cultured for 48 h after the start of treatment. Graphs represent fold change from mock-transfected or DMSO-treated H69 cells. **(E–F)** Graphs are representative experiments from three independent repeats. The significance of differences between **Mix CT** and **Mix KD** or **Mix CT** and **Mix ATN-161** conditions was determined by Student’s *t*-test (^**+**^, *p* < 0.05, ^**++**^, *p* < 0.01).

As a central EMT regulator, ZEB1 is overexpressed in various cancer types including lung, breast and colon cancer and plays a critical role in EMT and tumorigenicity (Zhang et al., 2015). ZEB1 can act as both, a repressor of epithelial genes, and also a transcriptional activator of genes promoting tumorigenic properties such as stemness, proliferation, chemoresistance, and metastasis. As an activator, ZEB1 forms a complex with YAP1 to promote cancer progression (Lehmann et al., 2016; Feldker et al., 2020). Our microarray analysis revealed that YAP1 and its downstream targets are indeed upregulated in H69 cells during direct contact with fibroblasts (Supplementary Table S2). We confirmed that YAP1 and the downstream targets of ZEB1/YAP1, such as CYR61, CTGF and AXL are highly upregulated (Figures 5A, B). This mechanism was conserved in H209, a second SCLC cell line we used to confirm our results (Supplementary Figure S4). YAP1 targets play critical roles in EMT and cancer metastasis by remodeling the microenvironment and inducing an aggressive phenotype in cancer cells (Lehmann et al., 2016; Feldker et al., 2020). For example, CTGF is associated with metastasis and drug resistance (Kang et al., 2003; Lai et al., 2011; Shen et al., 2021). AXL is an oncogenic receptor tyrosine kinase that promotes survival, proliferation, migration, EMT and metastasis (Gjerdrum et al., 2010; Xu et al., 2011; Paccez et al., 2014). CYR61 as well is associated with tumor aggressiveness, therapy resistance, and metastasis (Lai et al., 2011; Saglam et al., 2014; Habel et al., 2019). Thus, by upregulating both ZEB1 and YAP1, cancer cells are triggering a program that facilitates cancer progression and invasion while still maintaining epithelial markers. YAP1 translocation to the nucleus upon Hippo pathway inactivation is an important tumor progression mechanism. YAP1 nuclear translocation is regulated by its dephosphorylated status (Shibata et al., 2018). Our results though suggest that YAP1 is mainly regulated at the transcription level in H69 cells in contact with fibroblasts. Nontheless, YAP1 was present in high levels in the nucleus of H69 cells in direct contact with fibroblasts (Supplementary Figure S5, Mix vs. CT), suggesting that indeed YAP1 is translocated to the nucleus and acts as a transcription factor for EMT markers. cAMP response element-binding protein (CREB) transcription factor is a positive regulator of YAP1 transcription (Zhang et al., 2012; Wang et al., 2013; Muranen et al., 2016) and an integrin alpha 5 (ITGA5)/FAK/CREB signaling axis was described to enhance YAP1 transcription in breast cancer (Zhang et al., 2020). On the other hand, integrin-α5β1 (ITGA5/ITGB1) on cancer cells bind fibronectin assembled on the surface of fibroblasts (Miyazaki et al., 2019; Miyazaki et al., 2020). Interestingly, ITGA5 and ITGB1 were both upregulated in our microarray analysis in H69 cells after direct interaction with fibroblasts and are part of the PPI cluster 2 (Figures 3B, C). Thus, upregulation of ITGA5 and ITGB1 and binding to fibronectin could be one of the mechanisms responsible for CREB activation of YAP1 transcription (Figures 5C, D). To test this hypothesis, we depleted ITGA5, ITGB1 and CREB from H69 cancer cells using siRNA duplexes prior to direct co-culture with fibroblasts. Following co-culture for 48 h, the levels of YAP1 and its target CYR61 were determined in cancer cells. We observed a significant 5 to 6- fold reduction in YAP1 mRNA levels in H69 cells depleted for either ITGA5 or ITGB1 as compared to non-targeting control conditions (Figure 5E, Mix ITGA5 KD and Mix ITGB1 KD vs. Mix CT). In addition, treatment with *ATN*-*161* (Ac-PHSCN-NH2), a novel small peptide antagonist of integrin α5β1, complemented our siRNA studies and significantly reduced YAP1 and CYR61 mRNA levels in H69 cells in direct contact with fibroblasts (Figure 5F, Mix CT vs. Mix ATN-161). Similarly, we detected a 2-fold reduction of YAP1 and CYR61 levels when H69 cells were depleted for CREB (Figure 5E, Mix CREB KD). Together, these results demonstrate that ZEB1/YAP1 axis in cancer cells potentially functions downstream of ITGA5/ITGB1 interaction with fibronectin on the surface of fibroblasts. Whether integrins play any role in the cancer cell attachment to the fibroblasts, or are upregulated subsequent to the initial interaction, further amplifying the activation loop, are important questions to address. Nontheless, targeting ITGB1 and ITGA5 by siRNA or ATN-161 inhibition did not result in significant loss of H69 attachment, supporting the second possibility.

### Paracrine regulation of EMT in cancer cells

**FIGURE 6:**
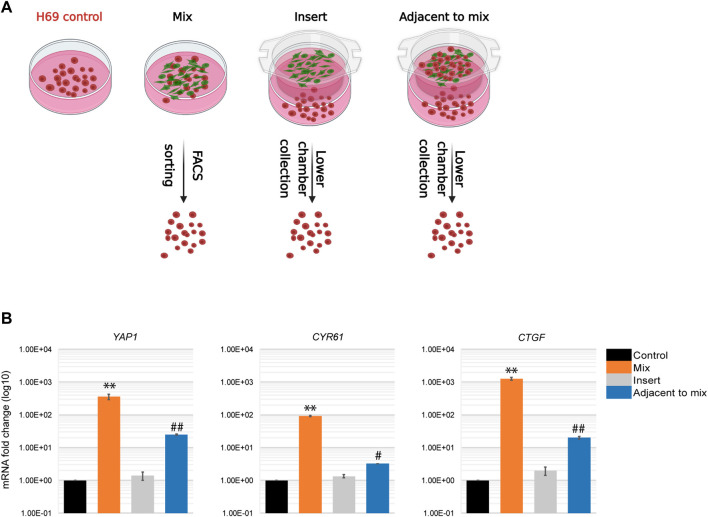
Paracrine regulation during cancer cells-fibroblasts direct interaction. **(A)** Experimental set-up for paracrine regulation of neighboring cancer cells. **Control**, H69 cells were grown in a separate dish. **Mix**, cells were co-cultured in direct contact before FACS sorting. **Insert**, H69 cells were grown in a dish separated from fibroblasts in the insert. **Adjacent to mix**, H69 cells were grown in a dish separated from the mixed cancer cells-fibroblasts co-culture in the insert. Cells were cultured in their respective conditions for 72 h before RNA extraction. **(B)** Relative mRNA levels were determined by qPCR for *YAP1* and its targets *CTGF* and *CYR61*. The significance of differences between **Control** and **Mix** conditions was determined by Student’s *t*-test (**, *p* < 0.01). The significance of differences between **Control** and **Adjacent to mix** conditions was determined by Student’s *t*-test (^#^, *p* < 0.05, ^##^, *p* < 0.01).

An alternative explanation for the distinct phenotypes we observed in cancer cells upon direct and indirect contact with fibroblasts is that the paracrine responses in these populations are different. To investigate this possibility, we altered the experimental design to monitor the response of cancer cells that are in close proximity to a mixed cell population but are not themselves involved in the direct interaction (Figure 6A, Adjacent to mix). To this end, a mixed population of cancer cells and fibroblasts were co-cultured in an insert on top of H69 cancer cells alone. The H69 cancer cells in the lower compartment do not contact the fibroblasts but can presumably respond to the diffusing factors released by the CCD8-H69 mixed population in the upper chamber. Following 72 h incubation H69 cells from all four conditions were evaluated for mRNA changes (Figure 6A, CT vs. Mix vs. Insert vs. Adjacent to mix). YAP1 and its targets were significantly upregulated in H69 cells in “Adjacent to mix” condition compared to the “Insert” condition in which fibroblasts alone were cultured in proximity to cancer cells (Figure 6B, Adjacent to mix vs. Insert). These results suggest that while the paracrine milieu is distinct under conditions that allow direct contact between cancer cells and fibroblasts, is however insufficient to drive the robust responses that direct contact induces in cancer cells. Thus, we speculated that in a tumor setting, a portion of cancer cells that contact the fibroblasts in the TME and undergo significant reprogramming can “educate” neighboring cells towards a more invasive phenotype.

### EMT reprogramming in cancer cells by fibroblasts is long-lasting but transient

**FIGURE 7:**
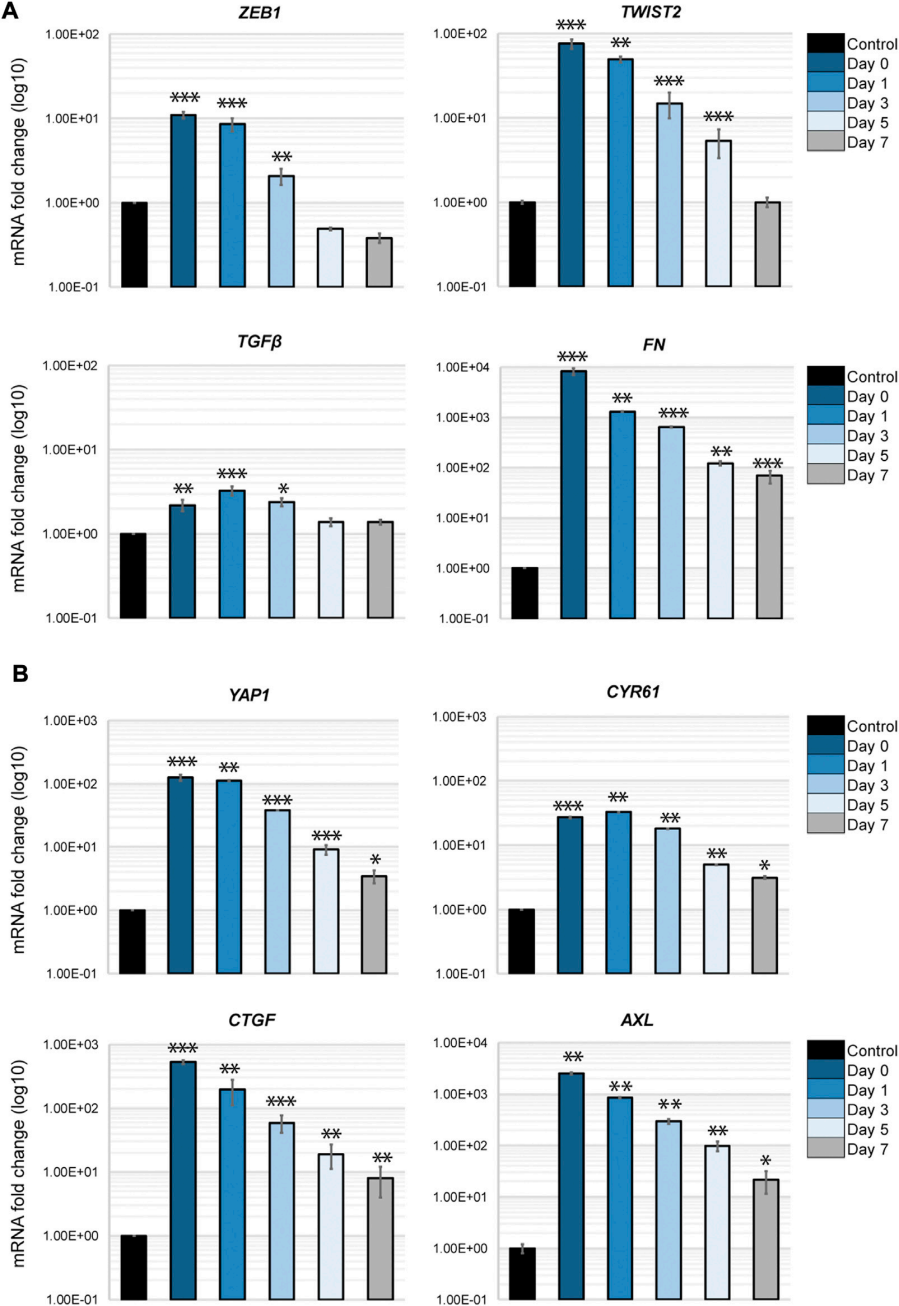
Phenotypic recovery in cancer cells after direct interaction. H69 were cultured alone (**Control**) or with fibroblasts for 72 h as described. H69 cells co-cultured in direct contact with CCD8 fibroblasts were FACS sorted. After sorting cancer cells were left to recover for determined amounts of time. **Day 0** is the day of sorting. **Day 1**, **Day 3**, **Day 5**, and **Day 7** are the times the H69 cells were left to recover in culture after separation from fibroblasts. Relative mRNA levels for EMT markers **(A)** and YAP1 pathway genes **(B)** were determined by qPCR. The significance of differences was determined by Student’s *t*-test (*, *p* < 0.05, **, *p* < 0.01, ***, *p* < 0.001).

An important aspect of the EMT in cancer cells is its plasticity. The dynamic transition between epithelial phenotypes (E), mesenchymal phenotypes (M) and hybrid E/M phenotypes plays a fundamental role in metastasis and cancer progression. We sought to determine if the transcriptional changes occurring in H69 cells in direct contact with fibroblasts are reversible in our model system. Thus, we monitored EMT markers expression in cancer cells that were in direct interaction with fibroblasts post-separation to assess the phenotype plasticity. The EMT-TFs, ZEB1 and TWIST2, as well as TGFβ1 revert to their original levels by day 7 after the contact was disrupted (Figure 7A, ZEB1, TWIST2 and TGFβ). However, mesenchymal markers that were strongly upregulated still displayed high levels of mRNA 7 days post-separation (Figure 7B, FN1). This was also true for both YAP1 and its targets (Figure 7B). Therefore, we conclude that i) transcriptional changes developed in cancer cells during the direct interaction with fibroblasts are transient and gradually revert to the original phenotype once the direct interaction is disrupted, and ii) the strength of the response dictates the time to recovery. However, we cannot exclude the possibility that longer contact with fibroblast changes the time reversibility of the phenotype in cancer cells, as epigenetic changes might occur during prolonged interaction (Dong et al., 2020). This aspect of EMT plasticity is currently under investigation.

### Discussion

The complex interaction between cancer cells and fibroblasts plays a critical role in tumor progression by promoting EMT in cancer cells and ECM deposition. The “seed and soil” hypothesis proposed over a century ago by Stephen Paget (Fidler, 2003) is being constantly revisited and appended by new evidence of how tumor cells and fibroblasts interact and manipulate each other. While paracrine communication between the stroma and the tumor is well established, less is known about how direct physical contact between cancer cells and fibroblasts mediates EMT in cancer cells and ECM remodeling. Here, we show that direct contact between cancer cells and fibroblasts induces a plastic hybrid EMT phenotype in cancer cells centered on cellular reprogramming that favors cell interaction with the ECM and invasion, which are key early steps in metastasis.

We established a model in which SCLC H69 suspension cells are grown in direct or indirect contact with adherent lung fibroblasts. We observed that lung cancer cells attach to the surface of normal lung fibroblasts and seize them for cell migration (Figure 1A). The extended duration of the interaction allows cancer cells to migrate together with fibroblasts either on their cell surface or frequently on long protrusions sent by the fibroblasts. The pulling force exerted by the fibroblasts on cancer cells during migration results in the dissociation of cancer cells from the suspension clusters and several rounds of stretching which could trigger mechano-transduction pathways (Labernadie et al., 2017).

Next, gene ontology analysis and PPI analysis revealed significant enrichment and clustering of genes involved in cell-matrix interaction and ECM remodeling (Figures 2, 3). EMT-inducing factors such as TGFβ1 and IL6 were upregulated in H69 following direct interaction (Figures 2A,C, cluster 1), suggesting that multiple concomitant pathways are activated in cancer cells in our system. Indeed, both TGFβ1 and IL6 have been shown to promote EMT and cancer cells metastatic potential (Wang et al., 2017; Deshmukh et al., 2021).

We further show that cancer cells in direct interaction with fibroblasts undergo transcriptional changes and display a hybrid E/M phenotype in which cells maintain high E-cadherin expression while at the same time upregulating several EMT-TFs and EMT markers (such as ZEB1, ZEB2, TWIST2, vimentin, fibronectin) (Figure 4). YAP1, a major regulator of EMT and activator of mesenchymal genes together with ZEB1 (Lehmann et al., 2016; Feldker et al., 2020), was upregulated in our experimental set-up together with its target genes (Figure 5A). We demonstrate that integrins ITGB1 and ITGA5, which are also upregulated in cancer cells during direct contact with fibroblasts, are involved in YAP1 transcriptional regulation *via* the CREB pathway (Figures 5C, E). The direct interaction results in the secretion of paracrine factors that in turn can induce EMT in neighboring cells (Figure 6). Finally, we show that the partial EMT phenotype we observe is reversible, and differentially regulated genes in cancer cells return to their original levels after dissociation from fibroblasts (Figure 7).

Our new findings are significant for several reasons. While complementing previous cancer cells/fibroblasts co-migration *in vitro* models (Labernadie et al., 2017; Miyazaki et al., 2020), our SCLC-lung fibroblast model highlights the critical role the direct contact between cancer cells and fibroblasts plays in EMT intermediate phenotypes and plasticity. An earlier *in vivo* model of lung metastasis demonstrated that cancer cells co-traveling with stromal fibroblasts increase the efficiency of brain metastasis and have a viability and growth advantage (Duda et al., 2010). Similarly, co-migration of colon carcinoma cells with CAFs enhances liver metastasis through a TGFβ-induced mechanism (Gonzalez-Zubeldia et al., 2015). Our data enhances the understanding of these mechanisms by providing critical evidence of how direct interaction modulates EMT plasticity in cancer cells. We demonstrate that only after direct contact with fibroblasts the cancer cells start an extensive reprogramming process that results in the upregulation of EMT inducers, such as TGFβ1, and subsequent regulation of genes facilitating interaction with the ECM and invasion. Critically, cancer cells acquire the capacity to synthesize their own ECM (Table 2) which enhances their capacity for invasion and survival (Pickup et al., 2014). While the direct interaction is the first step of this process, conditioned media from mixed cells can influence nearby cancer cells and induce activation of a similar program, albeit at lower levels (Figure 6). This is suggestive of a secondary surge of soluble factors from both cancer cells and fibroblasts that would reinforce the EMT phenotype in neighboring cancer cells, thus amplifying the EMT activation loop. Although in our microarray analyses we detected very few genes with significant changes in fibroblasts, additional gene analysis including markers of CAFs revealed that normal fibroblasts undergo transcriptional changes following direct interaction as well, and transition to a CAF-like phenotype (data not shown). The more moderate effect in fibroblasts is possible due to the timing of interaction or the number of cancer cells used in our assay. Additional experimental work is underway to substantiate these original findings.

One critical EMT-TF that was highly upregulated in our model was YAP1. This is significant for a couple of reasons: i) as a transcription activator, YAP1 overexpression promotes EMT in multiple cancers (Akrida et al., 2022), and ii) YAP1 enhances chemoresistance in a subpopulation of SCLC (Pearsall et al., 2020; Wu et al., 2021). Nuclear translocation of YAP1 following Hippo pathway inactivation has been extensively studied. Our results however revealed that YAP1 is transcriptionally activated through an ITGA5/ITGB1/CREB pathway after interaction with fibroblasts in cancer cells. We propose that a similar scenario might occur *in vivo* when a subpopulation of SCLC in direct contact with fibroblasts upregulates YAP1 and becomes resistant to therapy. Indeed, although rarer than the other SCLC subpopulations driven by ASCL1, NEUROD1, and POU2F3 (Rudin et al., 2019), the YAP1 subpopulation is nontheless an aggressive SCLC subpopulation (Pearsall et al., 2020). Because SCLC subtypes are dynamic (Ireland et al., 2020), YAP1 lineage can emerge during disease progression possibly through interaction with fibroblasts.

Together with YAP1 several EMT-TFs (ZEB1, ZEB2, TWIST2) are upregulated in cancer cells after contact with fibroblasts (Figure 3A). Yet, E-cadherin mRNA and protein abundance did not decrease (Figure 3B), indicating that epithelial and mesenchymal markers coexist in hybrid phenotypes when specific EMT-TFs are upregulated. Several factors could potentially explain the hybrid EMT phenotype in our system: i) cell specificity determines the EMT-TFs acting as repressors on epithelial genes; ii) EMT-TFs distribution in specific “repressor” and “activator” complexes is critical to expression changes of epithelial and mesenchymal genes; iii) upregulation of epithelial lineage transcription factors such as OVOL1; iv) hybrid phenotypes exist on a continuum and multiple fully activated pathways are required for complete transition. First, the tissue specificity of EMT-TFs has been demonstrated previously (Stemmler et al., 2019). For example, SNAIL triggers metastasis in breast cancer (Tran et al., 2014), whereas ZEB1 favors metastasis in pancreatic cancer (Krebs et al., 2017). Second, the repressor and activator functions of EMT-TFs could be key to the balance of markers in the hybrid EMT phenotypes. ZEB1 has been shown to act not only as a major repressor of epithelial genes but also as a critical activator of mesenchymal genes when in complex with YAP1 (Lehmann et al., 2016). A curated list of YAP1/ZEB1 gene targets analysis showed that multiple of these genes are upregulated in our system as well (Supplementary Table S2). Thus, the direct interaction between cancer cells and fibroblasts induces upregulation of both ZEB1 and YAP1, which in turn promotes a transcriptional reprogramming towards a more aggressive hybrid EMT phenotype. In breast tumors, for example, high expression of the ZEB1/YAP1 target genes results in a significantly shorter relapse-free and overall survival (Feldker et al., 2020). Third, co-expression of epithelial lineage transcription factors such as OVOL1 could counterbalance the effects of EMT-TFs repressor functions. Although OVOL1 did not meet the criteria to be included in the microarray gene signature, the qPCR analysis showed that it is highly upregulated in cancer cells in direct contact with fibroblasts (Supplementary Figure S3). A mathematical model previously predicted that OVOL transcription factors can stabilize a hybrid E/M phenotype (Jia et al., 2015) which is consistent with OVOL acting as a “molecular brake on EMT” (Watanabe et al., 2014). Finally, our model describes intermediate EMT phenotypes 3 days-post direct contact. If prolonged contact would ultimately result in cadherin switch and loss of epithelial markers, as detailed previously in TGFβ1 treatment (Deshmukh et al., 2021), remains to be determined.

Lastly, we show that the transcriptional changes in cancer cells are transient and gene expression slowly diminishes to pre-contact levels (Figure 7). This plasticity is potentially critical in explaining the MET transition occurring in secondary tumors after invasion from the primary site. Whether longer periods of contact result in extended time to gene expression recovery is currently under investigation. Additional epigenetic changes could occur during prolonged contact that induce a more stable EMT phenotype. The duration of interaction with the ECM thus becomes critical in phenotypical switches between different hybrid EMT states, their invasive potential and response to therapy. Altogether, our findings shed new light on the cancer cell-fibroblasts interaction and open new avenues of investigations into the complex EMT/MET switch and cancer cells plasticity.

## Materials and methods

### Cell culture conditions

CCD8 (CCL-201), H69 (HTB-119) and H209 (HBT-172) cells were obtained from ATCC and grown in RPMI media (Genesee) supplemented with 10% FBS. Cells were cultured at 37°C in a 5% CO2 incubator. All cell lines were authenticated by short tandem repeat (STR) profiling. All cell lines were tested for *Mycoplasma* contamination. Two types of co-cultures were performed. First, direct contact co-culture, which we refer to as “Mix”. The second type of co-culture is an indirect co-culture in which cancer cells and fibroblasts are separated by a cell-culture 1.0 µm pore insert. We refer to this co-culture condition as “Insert”. For both co-cultures the ratio of fibroblasts:cancer cells was 1:6. For both conditions, fibroblasts were seeded 24 h prior to addition of H69 cells to allow them to adhere.

### Live imaging

H69 cancer cells and CCD8 fibroblasts were labeled with PKH26 Red Fluorescent Linker Kit and PKH67 Green Fluorescent Linker Kit respectively (Sigma) according to the manufacturer’s instructions prior to co-culture. IncuCyte ZOOM was used to acquire images every hour for a total of 72 h.

### Cell labeling and FACS sorting

H69 cancer cells and CCD8 fibroblasts were labeled with PKH26 Red Fluorescent Linker Kit and PKH67 Green Fluorescent Linker Kit respectively (Sigma) according to the manufacturer’s instructions prior to co-culture. Shortly, H69 and CCD8 cells were collected, wash in serum free media twice and resuspended in 1 mL PKH diluent. The PKH26 and PKH67 dyes were diluted separately in PKH diluent (4 µL dye/1 mL diluent). Cells and dye were mixed and incubated for 5 min at room temperature. Staining was stop by addition of 10 mL of RPMI with 10% FBS. Cells were washed 2 more times with complete media before plating in single culture, insert or mixed culture. Following 72 h of co-culture, mixed cells were detached with EDTA and PBS, washed, and sorted according to their fluorescent marker using Bigfoot Spectral Cell Sorter (Invitrogen).

### Affymetrix microarrays

Microarray analysis was performed in the Research Core at LSU Health-Shreveport. RNA quality was determined with the Agilent TapeStation RNA assay (Agilent) and RNA quality was assessed with the Qbit Broad Range RNA assay (Invitrogen). RNA samples were processed and labeled for hybridization according to the standard GeneChip WT PLUS Reagent Kit Manual Target Preparation for GeneChip Whole Transcript (WT) Expression Arrays protocol. Approximately 250 ng of fragmented, biotin-labeled sense-strand ss-cDNA were hybridized to Affymetrix GeneChip Clariom D Human Arrays. The original contributions presented in the study are publicly available. GEO accession number is: GSE224873.

### Microarray analysis

Pixel intensity measurement, feature extraction, data summarization, normalization, and differential gene analysis were performed in Transcriptome Analysis Console (TAC) version 4.0. Arrays were normalized using the SST-RMA (Signal Space Transformation Robust Multi-Chip Analysis) algorithm, consisting of background adjustment, quantile normalization, and summarization. Expression analysis settings are as follows: Gene-level Fold Change < −2 or >2; Gene Level *p*-value <0.05; Anova Method: ebayes; a probeset (gene/exon) is considered expressed if > 50% samples have DABG values below DABG threshold (DABG <0.05).

### Gene functional analysis

Gene Ontology (GO) analysis associated with the DEGs was performed using the Database for Annotation, Visualization and Integrated Discovery (DAVID), with higher enrichment score signifying more functional enrichment (Jiao et al., 2012; Sherman et al., 2022) (https://david.ncifcrf.gov). Default values were used for functional annotation (Count: 2, EASE: 0.1). KEGG pathways analysis was completed using the “Pathways” software option in DAVID.

### Protein-protein interaction network (PPI) analysis

STRING (https://string-db.org) database of known and predicted protein-protein interactions (Szklarczyk et al., 2021) was used to build the Protein-Protein interaction (PPI) network of DEGs. The DEGs were mapped to the STRING database and known and predicted associations were scored and integrated. The “Minimum required interaction score” was set to ***high confidence*** (***0.700***). The DEGs were further analyzed using MCODE app in CYTOSCAPE software (https://cytoscape.org). The following parameters in MCODE were used for clustering: *Network Scoring*: Degree Cutoff: 2; *Cluster Finding*: Haircut Mode; Node Score Cutoff: 0.2; K-Case: 2; Max. Depth: 100.

### RNA extraction and quantitative PCR

RNA was extracted from cancer cells and fibroblasts using the RNeasy Kit from Qiagen. Total RNA and first-strand cDNA synthesis was performed using TaqMan Gene Expression Cells-To-Ct Kit (ThermoFisher) as previously described (Dragoi et al., 2014; Abshire et al., 2016). mRNA levels were determined by quantitative real-time PCR using the universal ProbeLibrary (Roche, Life Science) and LightCycler 480 Probes Master (Roche, Life Science) or PowerUp SYBR Green Master Mix (Thermo Fisher). For the LightCycler 480 Probes Master the thermal cycling was carried out using a LightCycler 96 instrument (Roche Diagnostics) under the following conditions: 95°C for 5 min and 40 cycles at 95°C for 10 s and 60°C for 25 s. For the PowerUp SYBR Green (for ITGA5 and ITGB1) the thermal cycling was carried out under these conditions: preamplification: 95°C for 10 min; amplification: 40 cycles at 95°C for 10 s, 60°C for 10 s and 72°C at 10 s; melting: 95°C for 10 s, 65°C for 60 s and 97°C for 1 s. Relative quantification was performed using 2^−ΔΔCT^ method. Gene expression was normalized to GAPDH as reference gene. The fold increase is represented as relative values to the **Control** condition. A complete list of primers and probes used in the study are presented in Supplementary Table S10.

### RNAi assays

Cells were reverse transfected with Dharmafect1 (Dharmacon) and a SMARTpool of the four individual siRNA silencing duplexes (12.5 nmol/L each, 50 nmol/L total). All siRNA duplexes were purchased from Horizon/Dharmacon: CREB1 (M-003619-01-0005), ITGB1 (M-004506-00-0005), ITGA5 (M-008003-02-0005). Before co-culture with fibroblasts, H69 cells were transfected in 6-well plates for 48 h. Following knock-down, H69 cells were washed with PBS to remove the siRNA duplexes prior to co-culture.

### Integrins inhibition assay

*Integrin* α5β1 *inhibition* was carried out using *ATN-161* (Ac-PHSCN-NH2), a small peptide antagonist of *integrin* α5β1. After the initial 24 h of co-culture, ATN-161 (10 µm final concentration) was added in the culture media before allowing the co-culture to continue for another 48 h. DMSO diluted in RMPI was used as control condition in the mixed cells population. FACS sorting after treatment was performed as described.

### Western blot analysis

H69 cells FACS sorted after 3 days of co-cultured were immediately lysed in RIPA buffer prior to SDS-PAGE analysis and immunoblotting. For cyto/nuclear extraction the NE-PER Nuclear and Cytoplasmic Extraction Reagent Kit (Thermo Fisher) was used according to the manufacturer’s instructions. The primary antibodies used were E-cadherin (Clone 36; BD Transduction Laboratories), Actin (C-2; Santa Cruz), fibronectin (sc-69776, Santa Cruz), CTGF (sc-365970, Santa Cruz). The following antibodies were all purchased from Cell Signaling: ZEB1 (E2G6Y), ITGB1 (D6S1W), ITGA5 (D7B7G), phosphor-CREB (87G3), YAP1 (D8H1X), CYR61 (D4H5D), SNAIL (C15D3), AXL (C89E7), Lamin A/C (4C11) and tubulin (9F3). Secondary antibodies horseradish peroxidase (HRP)-anti-mouse and anti-rabbit (1:5,000) were from Jackson Laboratories.

### Statistical analysis

All experiments were performed at least three times. For qPCR analysis, some experiments were performed five-to-six times. Data are presented as representative graphs and images Western blots from repeats. The analysis utilized Student’s *t* tests to determine significance. Values of *p* < 0.05 were considered significant, values of *p* < 0.001 were considered highly significant.

## Data Availability

The data presented in the study are deposited in the GEO repository: GSE224873.
