# Supplementary figures and images for: Assessing the epithelial-to-mesenchymal plasticity in a small cell lung carcinoma (SCLC) and lung fibroblasts co-culture model
Source: Front Mol Biosci. 2023 Mar 3;10:1096326. doi: 10.3389/fmolb.2023.1096326 (PMC10022497; doi:10.3389/fmolb.2023.1096326)

Supplemental Figure 6

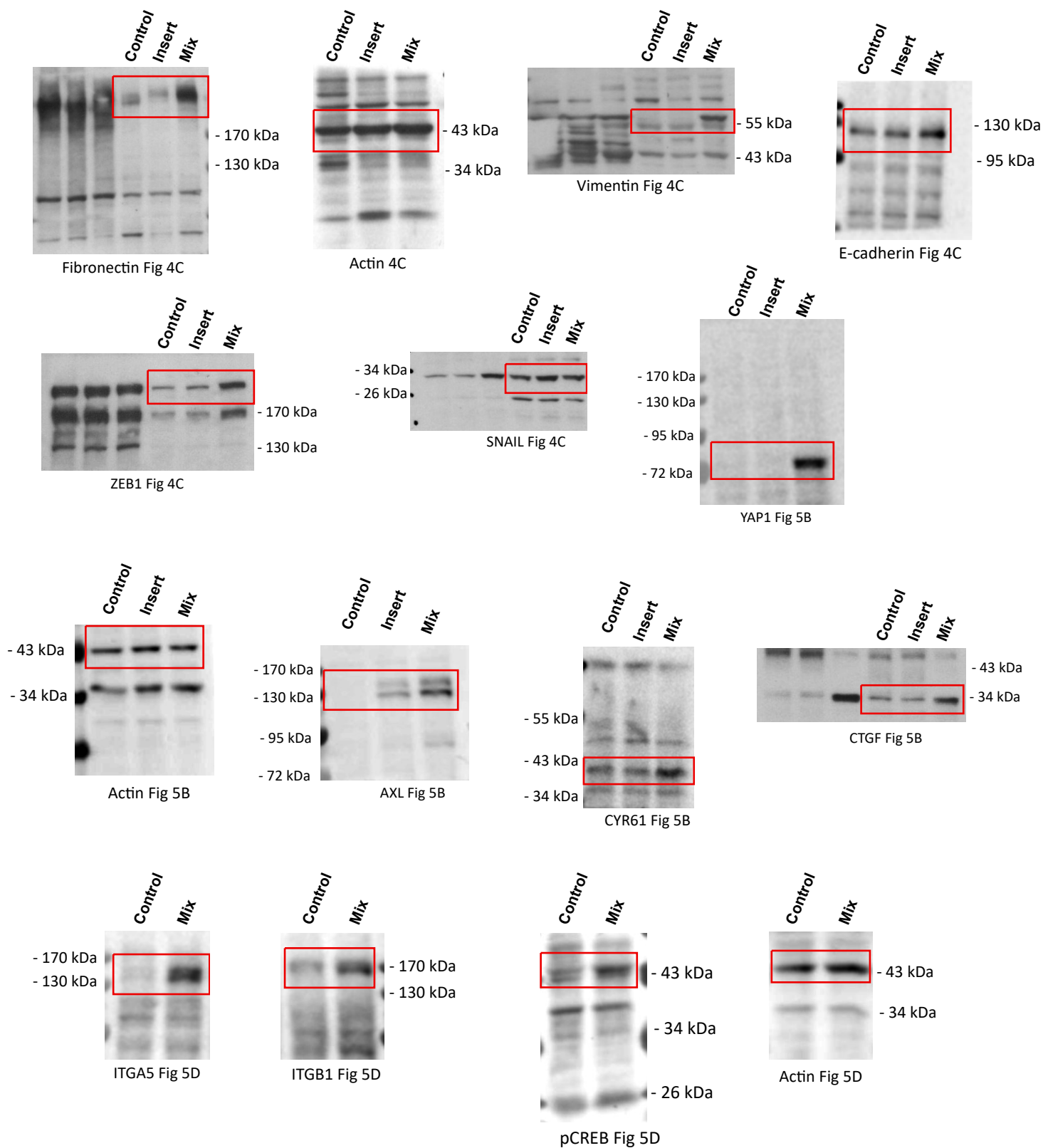

Supplement: Supplementary file 1 [file DataSheet7.PDF]

Supplemental Figure 1

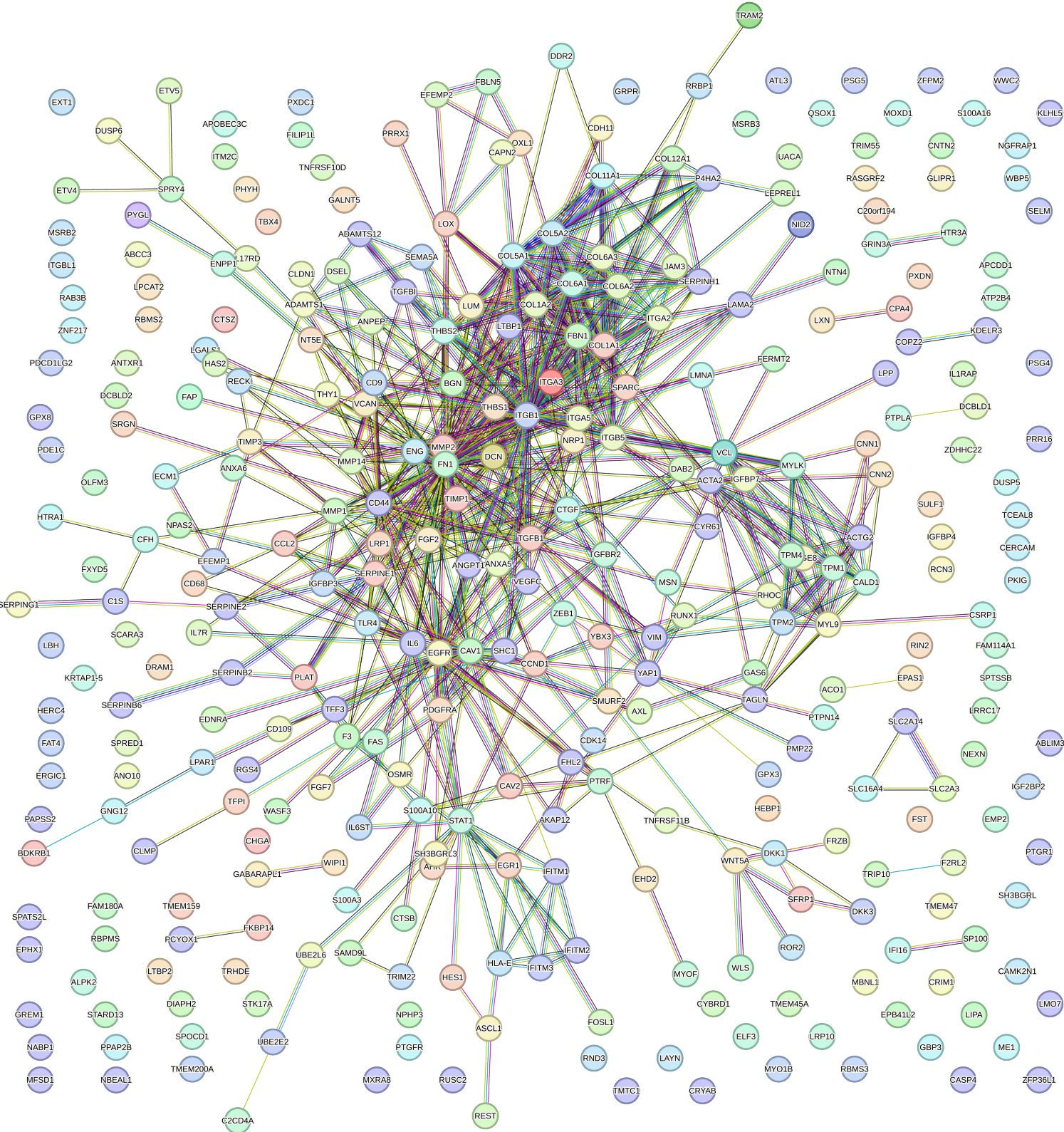

Supplement: Supplementary file 2 [file DataSheet2.PDF]

Supplemental Figure 3

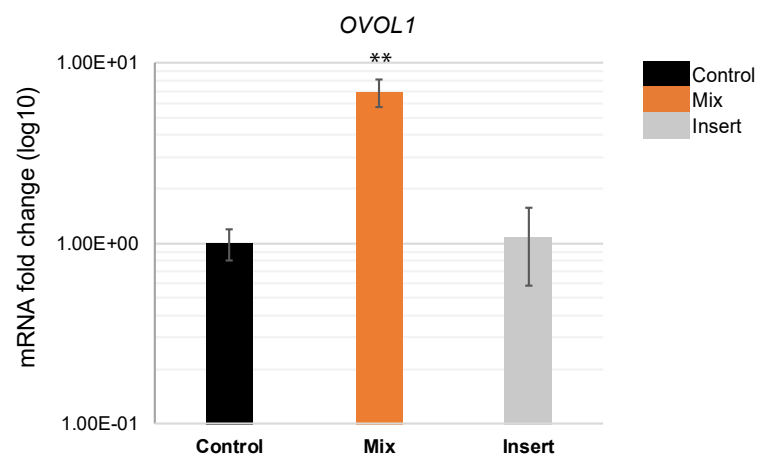

Supplement: Supplementary file 4 [file DataSheet4.PDF]

Supplemental Figure 5

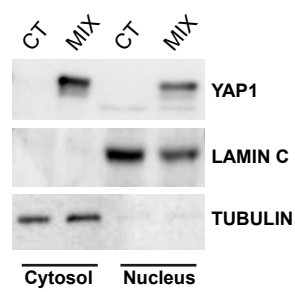

Supplement: Supplementary file 5 [file DataSheet6.PDF]

Supplemental Figure 2

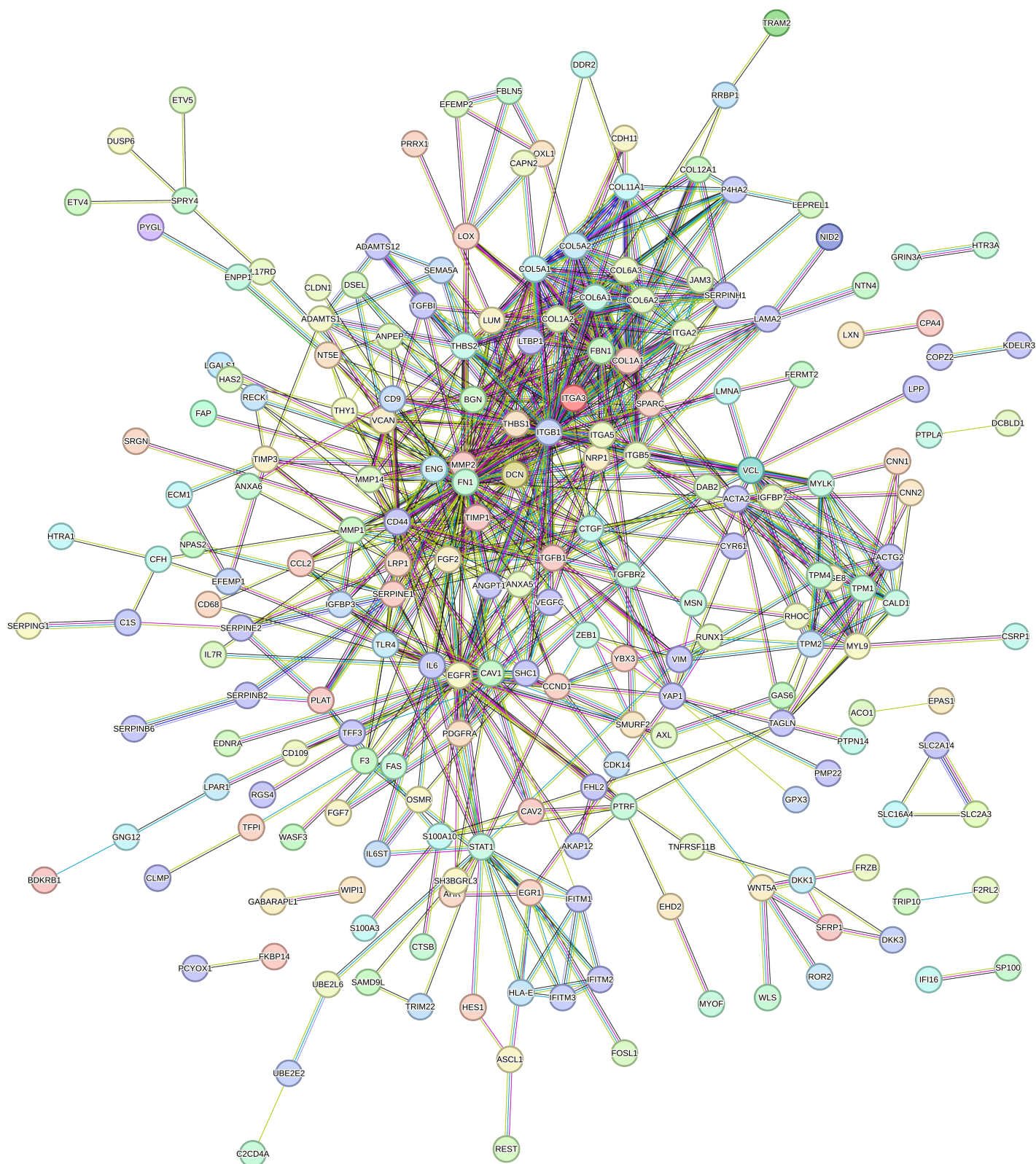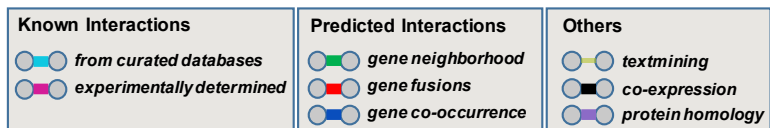

Supplement: Supplementary file 8 [file DataSheet3.PDF]

Supplemental Figure 4

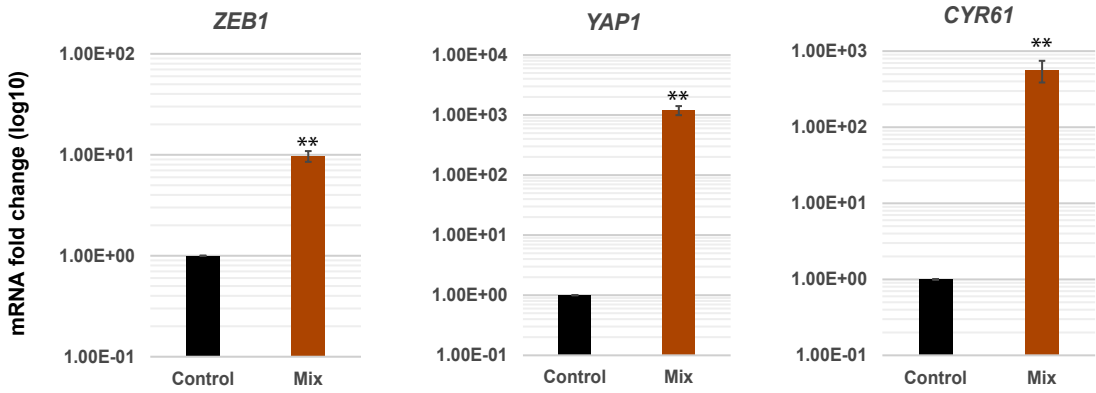

Supplement: Supplementary file 11 [file DataSheet5.PDF]
