# Supplementary material for: Assessing the epithelial-to-mesenchymal plasticity in a small cell lung carcinoma (SCLC) and lung fibroblasts co-culture model
Source: Front Mol Biosci. 2023 Mar 3;10:1096326. doi: 10.3389/fmolb.2023.1096326 (PMC10022497; doi:10.3389/fmolb.2023.1096326)
Supplement: Supplementary file 10 [file DataSheet1.PDF]

Supplemental Information

Supplemental Figure Legends

Supplemental Figure 1
PPI network of all DEGs in H69 Mix *vs* H69 insert. "Known interactions", "Predicted interactions" and "Others" are shown. All nodes were included in the PPI.

Supplemental Figure 2
PPI network of connected nodes in H69 Mix *vs* H69 insert. "Known interactions", "Predicted interactions" and "Others" are shown. Disconnected nodes were eliminated from the PPI.

Supplemental Figure 3
OVOL1 gene expression in H69 was quantified by qPCR in three different culture conditions. The significance of differences between **Control** and **Mix** conditions was determined by Student *t* test (**, $p<0.01$).

Supplemental Figure 4
ZEB1, YAP1 and CYR61 gene expression in H209 was quantified by qPCR in two different culture conditions. The significance of differences between **Control** and **Mix** conditions was determined by Student *t* test (**, $p<0.01$).

Supplemental Figure 5
YAP1 cytosolic and nuclear localization in H69 cells was determined after cyto/nuclear extraction in **Control** and **Mix** conditions. Tubulin was used as control for cytosolic fraction and lamin C was used as control for the nuclear fraction.

Supplemental Table 1.
Excel files for all genes, DEGs ($p<0.05$), and DEGs (FDR <0.1) from Affymetrix analysis for H69 Mix *vs* H69 CT.

Supplemental Table 2.
Excel files for all genes, DEGs ($p<0.05$), and DEGs (FDR <0.1) from Affymetrix analysis for H69 Mix *vs* H69 Insert.
"Top 10 genes" shows the highest scored DEGs.
"ZEB1/YAP1 genes and H69 genes" shows common genes in a curated list of known ZEB1/YAP1 targets (ref 59 and 60) and the DEGs in our experiment (H69 Mix *vs* H69 Insert).
"Gene expression for heat-maps" shows the DEGs in cluster 1 and cluster 2 with their respective expression level used Figures 3C and 3D.

Supplemental Table 3.
Excel files for all genes, DEGs ($p<0.05$) from Affymetrix analysis for H69 Insert *vs* H69 CT.

Supplemental Table 4.
Excel files for all genes, DEGs ($p<0.05$) from Affymetrix analysis for CCD8 Mix *vs* H69 CT.

Supplemental Table 5.
Excel files for all genes, DEGs ($p<0.05$) from Affymetrix analysis for CCD8 Mix *vs* H69 Insert.

Supplemental Table 6.
Excel files for all genes, DEGs ($p<0.05$) from Affymetrix analysis for CCD8 Insert *vs* H69 CT.

Supplemental Table 7.
Full GO analysis of BP, CC and MF of DEGs specifically regulated in H69 Mix *vs* H69 Insert.

Supplemental Table 8.
Full KEGG analysis of DEGs specifically regulated in H69 Mix *vs* H69 Insert.

Supplemental Table 9.
MCODE analysis and top clusters identified using DEGs identified in H69 Mix *vs* H69 Insert.

Supplemental Table 10.
QPCR primers and corresponding Universal Library Probes (Sigma-Aldrich) for the genes used in the study. SYBR green primers for ITGA5 and ITGB1 are listed at the bottom.

Movie 1 and 2
Representative movies from 72h live-acquisition of direct H69-CCD8 interaction. Images were acquired every hour in phase, red channel, and green channel. H69 cancer cells are in red, CCD8 fibroblasts are in green. Movies are from two independent experiments.
